# Molecular Genetics Solves the Conundrum of Two Brothers Affected With Proteinuria Coming With a Very Different Flavor: A Case Report

**DOI:** 10.1016/j.xkme.2025.100990

**Published:** 2025-03-11

**Authors:** Ludwig Haydock, Guillaume Dorval, Laurence Heidet, Bertrand Knebelmann

**Affiliations:** 1 Service de Néphrologie Adulte, Hôpital Necker-Enfants Malades, Assistance Publique, Hôpitaux de Paris (AP-HP): Université Paris Cité, Paris, France; Department of Medicine, Nephrology Research Group, Laval University, Quebec City, Quebec, Canada; 2 Laboratoire des Maladies Rénales Héréditaires, Inserm UMR 1163, Institut Imagine, Université Paris Cité, Paris, France; Service de Médecine Génomique des Maladies Rares, Hôpital Necker-Enfants Malades, Assistance Publique, Hôpitaux de Paris (AP-HP), Paris, France; 3 Laboratoire des Maladies Rénales Héréditaires, Inserm UMR 1163, Institut Imagine, Université Paris Cité, Paris, France; Service de Néphrologie Pédiatrique Centre de Référence Maladies Rénales Héréditaires de l'Enfant et de l'Adulte (MARHEA), Hôpital Necker-Enfants Malades, Assistance Publique, Hôpitaux de Paris (AP-HP), Paris, France; 4 Service de Néphrologie Adulte, Hôpital Necker-Enfants Malades, Assistance Publique, Hôpitaux de Paris (AP-HP): Université Paris Cité, Paris, France

**Keywords:** Proteinuria, receptors, cell surface, albuminuria, genetics, pathology, kidney tubules, proximal, proteinuria, glomerulosclerosis, focal segmental, β 2-microglobulin, urine, WT1, CUBN, Cubilin

## Abstract

Genetic testing is increasingly used to diagnose kidney diseases, proving cost-effective when performed on selected patients. We present the case of 2 brothers with proteinuria from a young age; one developed kidney insufficiency while the other maintained normal kidney function into late life. This case report investigates whether they inherited the same disease. The proband exhibited focal segmental glomerulosclerosis, with kidney function declining over time. Genetic analysis revealed heterozygous variants in *MYH9* and *WT1*. The *MYH9* variant was deemed nonpathogenic, whereas the *WT1* variant, associated with autosomal dominant nonsyndromic focal segmental glomerulosclerosis, likely contributed to the proband’s kidney insufficiency. However, this variant was absent in his brother, who also had proteinuria but preserved kidney function. Further analysis identified biallelic variants in *CUBN* in both brothers, suggesting a distinct cause of proteinuria for the brother with normal kidney function. This case illustrates 2 different genetic causes of proteinuria in siblings, highlighting the significance of genetic testing in differential diagnosis and personalized treatment. The findings emphasize the potential misdirection toward glomerular diseases of patients bearing *CUBN* variants and the general good prognosis associated with them. This case underscores the era of personalized medicine, in which genetic insights tailor treatment strategies for individual patients.

Genetic testing is becoming increasingly available and, when performed on selected patients, may be cost-effective in the investigation of kidney diseases.^1^ Herein we present the case of 2 brothers who presented with proteinuria at a young age. Intriguingly, one developed kidney insufficiency while the other maintained normal kidney function late in life. We investigated whether they inherited the same disease.

### Case Report

A 32-year-old White male (II-2) (Fig 1) presented with proteinuria (1g/day) and intermittent microscopic hematuria since childhood. Proteinuria was 1.1 g/day and estimated glomerular filtration rate (eGFR) at 90 mL/min/1.73m^2^; kidney ultrasound showed normal sized kidneys; on kidney biopsy 2 of 15 (18%) glomeruli were globally sclerotic without other significant abnormalities, no proliferation, no deposits, and important arteriolar hyalinosis and fibrous endarteritis of interlobular arteries. Immunofluorescence staining showed no immune deposits. In electron microscopy, one glomerulus out of 2 showed pseudo-epithelial proliferation with synechia, suggesting focal and segmental glomerulosclerosis (FSGS). His examination revealed no extrarenal features; in particular, the eye examination and audiogram were normal. The diagnosis of slowly progressing FSGS of unknown origin was suggested. At the age of 37, renin-angiotensin-system (RAS) blockade was initiated on the basis of high blood pressure and proteinuria (2.68 g/day) with normal albuminemia. Blood pressure normalized and proteinuria, composed mainly of albumin, stabilized around 2-3 g/day; urinary β-2 microglobulin (β_2_-M) was only slightly elevated (471 μg/L; ref value < 350 μg/L) and retinol binding protein was undetectable (< 0.6 mg/L). Kidney function declined by a mean of 1.5 mL/min/year since the age of 29. At last follow-up (62 years), eGFR was 33 mL/min/1.73m^2^ and proteinuria 2.7 g/day under maximal dose of sartan, eplerenone, and SGLT2 inhibitor (Fig 2).

**Figure 1:**
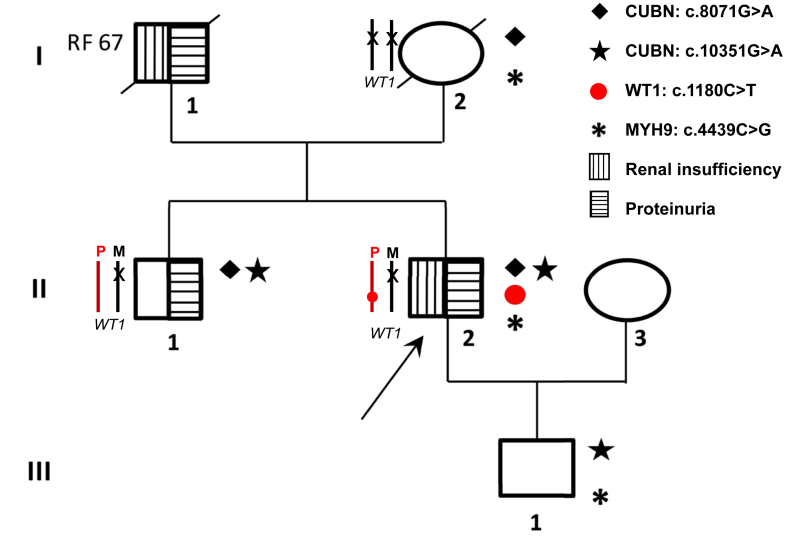
Pedigree of the family. To study the paternal or maternal inheritance of the variant c.1180C>T of the *WT1* gene identified in II-2 (in the absence of available paternal DNA), we studied the segregation of the c.1122A>G polymorphism in exon 7, identified in II-2 and located 58 bp downstream from the *WT1* c.1180C>T mutation. We were able to show that I-2 carried the c.1122A>G polymorphism in the homozygous state. This led to 2 hypotheses: (1) either the c.1180C>T variant arose de novo in the paternal allele of II-2, or (2) the c.1180C>T variant was inherited from I-1. Haplotypes from maternal origin are in black, X denoting the maternal A>G polymorphism; haplotype from paternal origin is in red. P, paternal; M, maternal.

**Figure 2:**
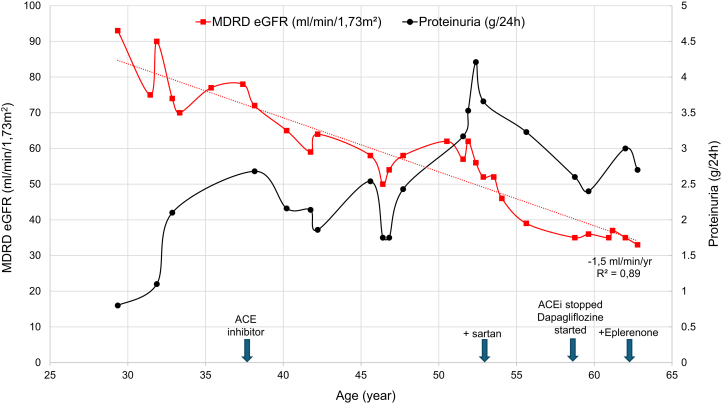
Proband (II-2); MDRD eGFR (red line)—proteinuria (black line)—treatment timeline (blue arrows). eGFR, estimated glomerular filtration rate.

Family history (Fig 1) revealed that his father (I-1) had proteinuria since a young age and reached kidney failure (KF) at the age of 67; no kidney biopsy was performed. His mother (I-2) was not known for any health problems and died at 93 without any kidney problem. His brother (II-1) had proteinuria around 500 mg/day since childhood, composed mainly of albumin, without hematuria. Urinary β_2_-M was slightly elevated (408 μg/L; ref value< 350 μg/L) and retinol binding protein was undetectable (< 0.6 mg/L). Kidney function was still strictly normal (eGFR at 80 mL/min/1.73m^2^) at the age of 74 (Fig 3). His son (III-1) had normal kidney function without proteinuria or hematuria at age 23.

**Figure 3:**
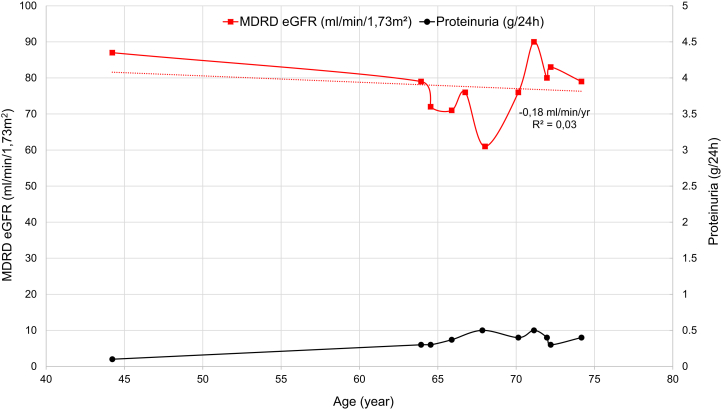
Proband’s brother (II-1); MDRD eGFR (red line)—proteinuria (black line). eGFR, estimated glomerular filtration rate.

#### Molecular Genetics Analysis

This familial renal phenotype prompted a targeted FSGS-associated genes panel analysis, performed in an accredited diagnostic laboratory (Table 1) in the proband. It revealed 2 heterozygous variants, one in *MYH9* and one in *WT1*.

**Table 1:** Focal Segmental Glomerulosclerosis (FSGS) and Subsequent Proteinuria Panels

Isolated Steroid-Resistant Nephrotic Syndrome
Autosomal recessive: *NPHS1*; *NPHS2*; *PLCE1/NPHS3*; *PTPRO*; *MYO1E*; *ARHGDIA*; *TTC21B*; *CRB2; MAGI2*; *PODXL*; *FAT1*; *CD2AP*; *DGKE*; *EMP2*; *KANK2*; *KANK4*; *LAMA5*; *GAPVD1*; *ANKFY1*; *AVIL*; *APOL1* (allele G1/G2) X-Linked: *TBC1D8B* Autosomal dominant: *TRPC6*; *ACTN4; INF2*; *PAX2*; *WT1*; *ANLN*
Syndromic steroid-resistant nephrotic syndrome
Autosomal recessive: *ALG1*; *ITGA3*; *LAMB2; NUP85*; *NUP93*; *NUP107; NUP160; NUP205*; *PMM2*; *SMARCAL1*; *SGPL1* X-Linked: *NXF5*; *GLA* Autosomal dominant: *LMX1B***;***MYH9*
Collagen type IV
Autosomal recessive/autosomal dominant: *COL4A3; COL4A4* X-Linked: *COL4A5*
Galloway-Mowat syndrome
Autosomal recessive: *WDR73*; *OSGEP; TP53RK; TPRKB; GON7; YRDC; WDR4; NUP133; NUP107* X-Linked: *LAGE3*
Mitochondrial dysfunction
Autosomal recessive: *MTTL1*; *COQ2*; *COQ6*; *COQ8B*; *PDSS2*
Proteinuria-causing genes
Autosomal recessive: *CUBN*; *CTNS*
X-Linked: *CLCN5*; *OCRL*

The variant in exon 32 of *MYH9* (NM_002473.6) (c.4439C>G; p.Ser1480Trp) has not been reported in the general population (gnomAD v4.1.0 database, [https://gnomad.broadinstitute.org/]), nor in patients with *MYH9*-related disease (HGMD-Pro [https://my.qiagendigitalinsights.com/bbp/] and ClinVar [https://www.ncbi.nlm.nih.gov/clinvar/] queried in July 2024) and was classified as variant of unknown significant (Table 2). Heterozygous variants in *MYH9* are associated with autosomal dominant steroid-resistant glomerular disease with intrafamilial variability in kidney disease severity, but also nearly always extrarenal features, notably macrothrombocytopenia, leukocyte inclusions, and sometimes hearing loss and cataracts. Very few cases of *MYH9-*related FSGS with normal platelet count at diagnosis have been described, but all showed intermittent macrothrombocytopenia during their follow-up.^2^ Our patient had repeatedly normal platelet count, no giant platelets, and no other extrarenal features, suggesting the *MYH9* variant was not involved in his kidney disease. Most importantly, it was found in both the proband’s healthy mother (I-2) and his son (III-1) but not in his brother (II-1). The lack of segregation of *MHY9* variant with proteinuria and renal insufficiency in the kindred strongly suggests that it is not pathogenic.

**Table 2:** Characteristics of the Identified Variant

Gene	NM_	Nucleotide change	Amino acid change	GnomAD (v4.1.0)	PolyPhen2 (HumVar)	SIFT	ACMG	ACMG
*MYH9*	NM_002473.6	c.4439C>G	p.Ser1480Trp	0	0.967 (probably damaging)	0.001 (Damaging)	PM2 PP2; PP3 BS4	3
*WT1*	NM_024426.6	c.1180C>T	p.Arg394Cys	5/1,614,142	1.000 (probably damaging)	0.000 (Damaging)	PM1; PM2 PP3	3
*CUBN*	NM_001081.4	c.8071G>A	p.Gly2691Arg	670/1,613,860	0.989 (probably damaging)	0.000 (Damaging)	PM1; PM2	3^a^
*CUBN*	NM_001081.4	c.10351G>A	p.Asp3451Asn	215/1,613,712	1.000 (probably damaging)	0.052 (Tolerated)	PM1; PM2 PP3	3^a^

SIFT, sorting intolerant from tolerant; ACMG, The American College of Medical Genetics and Genomics; VUS, variant of unknown significant.

a These variants were considered proteinuria-causing in this article even though they were strictly VUS according to the ACMG/AMP guidelines.

The variant in *WT1* (NM_024426.6) exon 7 (c.1180C>T; p.Arg394Cys) was also classified as variant of unknown significant (Table 2). It has been reported in gnomAD v4.1.0 database 3x10^−6^ but has also been reported in individuals with FSGS, notably in 2 brothers with autosomal dominant nonsyndromic FSGS.^3^ Heterozygous variants in *WT1* are associated with diverse phenotypes: (i) Denys-Drash syndrome with the triad of early-onset nephrotic syndrome with diffuse mesangial sclerosis, genital abnormalities, and Wilms’ tumor susceptibility, (ii) Frasier syndrome typically presenting with later-onset FSGS, genital abnormalities, and gonadoblastoma susceptibility, or (iii) autosomal dominant nonsyndromic FSGS, which could be the case for our patient.

The *WT1* variant was not found in his mother (I-2) thus was de novo or inherited from his father (I-1), for whom DNA was unavailable (Fig 1). It was absent in his son (III-1), but surprisingly, it was also absent in his proteinuric brother (II-1). Thus, the *WT1* variant could explain the proteinuria and renal insufficiency of our patient (II-2) and probably his father (I-1) but not the proteinuria of his brother (II-1). To shed light on this surprising finding, we expanded our panel to other proteinuria-causing genes (Table 1). We found that both brothers (II-1, II-2) carried 2 variants in the *CUBN* gene (NM_001081.4). The first variant (c.8071G>A; p.Gly2691Arg) in exon 52 is present in 4 × 10^−4^ gnomAD v4.1.0 population and has already been reported in *CUBN*-associated proteinuria individuals at a homozygous and compound heterozygous state.^4^^,^^5^ The second variant (c.10351G>A; p.Asp3451Asn) in exon 64, is present in 1.3 × 10^−4^ gnomAD v4.1.0 population and has never been reported in individuals affected with *CUBN*-related conditions in the literature. However, it is located in CUB26 domain, a highly suspected albumin binding site of cubilin.^6^ Both variants were classified as variant of unknown significant (Table 2). One of the 2 *CUBN* variants (c.8071G>A and p.Gly2691Arg) was found in the mother (I-2) and the other (c.10351G>A; p.Asp3451Asn) in his son (III-1), confirming the trans configuration of the 2 variants in our patient (II-2) and his brother (II-1). The biallelic *CUBN* variants probably contribute to the residual proteinuria under RAS blockade, eplerenone, and SGLT2 inhibitor of our patient (II-2) and could explain the preserved kidney function at age 74 despite proteinuria in his brother (II-1), who did not inherit the *WT1* variant.

## Discussion

This case illustrates 2 different genetic causes of proteinuria in 2 brothers, one associated with a benign condition and the other with progressive kidney disease.

Little is known regarding genotype-phenotype correlation in patients with *WT1*-related autosomal dominant nonsyndromic FSGS. In 2010, Benetti et al have described the first patients with this entity linked to a *WT1* missense variant in exon 9. Out of the 5 patients described, 3 reached KF at an age of 44, 46, and 69.^7^ In 2015, Hall et al reported another family with a missense variant in exon 9, in which 3 individuals reached KF at 17, 27, and 33 years.^8^ Finally, Ottlewski et al reported 2 brothers with the same exon 7 missense variant as our patient (II-2) one reached CKD4 at 61 years and the other KF at 61 years.^3^ Our case, added to those reported, illustrates the broad spectrum of *WT1*-related disease.

In contrast, *CUBN-*related proteinuria is typically moderate (0.5 to 2 g/day) and consists mainly of albumin, despite cubilin being primarily expressed in the proximal tubule. Tubular proteinuria occurs when low-molecular-weight proteins that are filtered through the glomerulus are not reabsorbed in the proximal tubule. It typically presents as high urinary β_2_-M and retinol binding protein, and a urinary albumin-to-protein ratio < 0.3. However, *CUBN*-related proteinuria is composed mainly of albuminuria and contains normal or only slightly elevated urinary β_2_-M and alpha-1 microglobulin (α1-M) despite being secondary to a tubular reabsorption defect (Table 3).^4^, ^5^, ^6^^,^^9^, ^10^, ^11^, ^12^, ^13^, ^14^, ^15^, ^16^, ^17^ This is probably explained by the normal expression of megalin, which binds these low molecular-weight proteins, while cubilin is critical for normal tubular uptake of albumin.^18^, ^19^, ^20^ Importantly, the higher-than-expected ratio of albumin/low-molecular-weight proteins seen in cubilin-related disease might misdirect the clinician toward a glomerular disease and prompt unnecessary treatment.

**Table 3:** Phenotype Associated With *CUBN* Variants

References	n	Age at Discovery of Proteinuria (Y)	Proteinuria (g/g)	Hypo-albuminemia	Hematuria	Increased	Increased	Age at Last FU (Y)	eGFR at Last F/U (mL/min/1.73m^2^)
						U α1-M	U β2-M		
Our case	1	Childhood	0.4 (0.1-0.5)	0/1	0/1	NA	1/1^a^	74	80
Yang et al^9^ (2024)	4	0.3	0.4 (0.3-0.6)	0/4	NA	0/4	1/4^a^	NA	NA
Shi et al^10^ (2023)	2	7.5 (7.0-8.0)	0.7 (0.6-0.7)	NA	NA	NA	NA	14 (14-15)	Normal
Ran et al^11^ (2023)	2	8.0 (4.5-12)	0.7 (0.6-0.8)	0/2	0/2	2/2^a^	NA	18 (14-22)	131 (123-140)
Cicek et al^5^ (2023)	6	7.3 (4.7–13)	0.8 (0.4-1.3)	0/6	0/6	NA	0/6	14 (5.0-20)	161 (152-178)
Madureira et al^12^ (2023)	2	5.0 (3.0-7.0)	1.6 (0.7-2.7)	0/2	0/2	NA	NA	9.5 (9.0-10)	144 (138-150)
Gan et al^13^ (2022)	2	2	0.4 (0.4-0.5)	0/2	NA	2/2^a^	0/2	8	Normal
Domingo et al^4^ (2022)	15	11 (0.8-44)	1.1 (0.2-2.1)^b^	1/15^c^	NA	NA	NA	22 (2.7-53)	114 (70- 180)
Yang et al^14^ (2022)	3	7.5 (5.0-11)	0.4 (0.4-0.5)	0/3	NA	NA	1/3^a^	8.0 (6.0-11)	155 (132-168)
Bedin et al^6^ (2020)	39	8.4 (0-36)	0.8 (0-2.4)	0/39	10/35	2/15^a^	1/14^a^	18 (0-71)	116 (73-172)
Jayasinghe et al^15^ (2019)	2	6.0 (4.0-8.0)	0.7 (0.6-0.8)	0/2	0/2	NA	NA	10 (8.0-12)	Normal
Schapiro et al^16^ (2019)	3	6.0 (0.8-12)	0.5 (0.3-0.9)	NA	3/3	NA	NA	NA	NA
Ovunc et al^17^ (2011)	2	4.5 (4.0-5.0)	0.6 (0.4-2.0)	0/2	NA	NA	NA	4.5 (4.0-5.0)	Normal
Total	83	8.2 (0-44)	0.81 (0-2.7)	1/78^c^	13/51	6/23^a^	4/30^a^	18 (0-74)	122 (70-180)

*Note:* N, number of patients; mean (min-max).

a Slightly increased.

b Excluding the patient with minimal change disease (MCD) (22g/g).

c MCD.

At the age of 74, the patient’s brother (II-1) still has a normal kidney function, making him the oldest patient reported with *CUBN*-related proteinuria. This, along with data from previous studies that showed no case of CKD stage 3 to 5 (Table 3), contribute to reassure nephrologists regarding the favorable long-term prognosis of this condition. However, the observation of podocyte cubilin expression rises the hypothesis of a specific glomerular function in albuminuria handling. Indeed, a few cases of *CUBN*-related proteinuria have been associated with FSGS lesions on kidney biopsy.^4^^,^^6^^,^^14^ It could have been related to other associated conditions, as in Domingo-Gallego et al and in our case (II-2), or it could be related to podocyte damage.^4^ Even if present in a small minority of described patients, these few FSGS observations suggest that these patients should benefit from long-term follow-up of their proteinuria and kidney function. Whether they should receive RAS inhibitors or not remains to be more firmly established.

In conclusion, this case illustrates the importance of genetic testing in the investigation of kidney diseases with a positive family history. Here, it allowed us to retro-phenotype our patient (II-2) and his brother (II-1), individualize their treatments, and rule out genetic disease in his son (III-1). Two brothers, 2 genetic diseases, 2 different prognoses, 2 different therapeutic approaches–that’s the era of personalized medicine.

## Declaration of generative AI and AI-assisted technologies in the writing process

During the preparation of this work the authors used ChatGPT only as an English-language corrector. After using this tool, the authors reviewed and edited the content as needed and take full responsibility for the content of the publication.
